# Hot Topics in Safety for Pediatric Anesthesia

**DOI:** 10.3390/children7110242

**Published:** 2020-11-20

**Authors:** Amanda N. Lorinc, Camila B. Walters, Hannah K. Lovejoy, Christy J. Crockett, Srijaya K. Reddy

**Affiliations:** Division of Pediatric Anesthesia, Vanderbilt University Medical Center, Nashville, TN 37232, USA; camila.walters@vumc.org (C.B.W.); hannah.lovejoy@vumc.org (H.K.L.); christy.crockett@vumc.org (C.J.C.); srijaya.k.reddy@vumc.org (S.K.R.)

**Keywords:** pediatric anesthesia, patient safety, perioperative safety events

## Abstract

Anesthesiology is one of the leading medical specialties in patient safety. Pediatric anesthesiology is inherently higher risk than adult anesthesia due to differences in the physiology in children. In this review, we aimed to describe the highest yield safety topics for pediatric anesthesia and efforts to ameliorate risk. Conclusions: Pediatric anesthesiology has made great strides in patient perioperative safety with initiatives including the creation of a specialty society, quality and safety committees, large multi-institutional research efforts, and quality improvement initiatives. Common pediatric peri-operative events are now monitored with multi-institution and organization collaborative efforts, such as Wake Up Safe.

## 1. Introduction

Anesthesiology has made great strides in patient safety over the past several decades, with mortality decreasing from 1:2500 to 1:13,000 [1,2,3]. Pediatric anesthesiology is inherently higher risk than adult anesthesiology due to the difference in physiology in children. The sub-specialty specializes in dealing with these differences. We aimed to describe the highest yield safety topics for pediatric anesthesia.

Pediatric perioperative safety events fall into major categories including airway, cardiovascular, and medication error events. Research analyzing the location of the anesthetic (operating room or OR versus non-operating room anesthesia or NORA) in determining perioperative risks, as well as care transition and handovers associated with transferring patients to and from the operating room from other areas in the hospital has been conducted. The Neonatal Intensive Care Unit (NICU) population is at particularly increased risk due to rapidly changing physiology and smaller margin of error. With the increased incidence of obesity, the field is encountering unique challenges in this population as well. Major research efforts to improve safety include the concern for neurotoxicity in small children. Several safety resources have been developed such as pediatric perioperative crisis and airway checklists through various pediatric anesthesiology organizations including the Society for Pediatric Anesthesia (SPA) and Wake Up Safe (WUS). In addition, WUS provides event data for analysis and dissemination and methods for quality improvement and enhanced patient safety.

## 2. Historical and Current Pediatric Perioperative Adverse Events

### 2.1. Historical Pediatric Perioperative Event Reporting

Historically, major studies that investigated pediatric perioperative adverse events include the following. Snyder et al. reported 23 cardiac arrests out of 57,600 anesthetics at Children’s Hospital Los Angeles from 1932 to 1952. The incidence increased from 1:2504 in the first 15 years to 1:1128 in last five years in conjunction with the change in practice from open drop delivery of ether to administration of volatile anesthesia via anesthesia machines. The hypotheses of these findings included dead space ventilation and resistance of the tubing leading to hypoxia and/or hypercarbia in small children [4].

Rackow et al. presented cardiac arrest data due to anesthesia from 1947 to 1958 at a frequency of 1:719 for patients under one year and 1:2326 for ages one through 12 versus 1:2580 in patients 13 years or older due to anesthesia and unknown causes [5]. Most common causes included anesthetic overdose, anoxia, inadequate ventilation/oxygenation, and aspiration. Eighty percent of these anesthetics were administered by residents with 2 ½ months of pediatric anesthesia experience. Almost all the remaining anesthetics were administered by attending anesthesiologists, with very few by nurses. In children who experienced a cardiac arrest, 16% had a full recovery, 16% had a partial recovery, and 68% died. By comparison, adults experienced 50% complete recovery after cardiac arrest.

In France, Tiret et al. gathered data on 40,240 pediatric (<15 years) anesthetics from 440 institutions from 1978–1982. They found 27 major complications (including death) and that the risk of complications was significantly higher in infants (4.3 per 1000) than in children (0.5 per 1000). The overall mortality rate was one in 40,000. Respiratory failure was the most common cause of complications in infants and tended to occur during the maintenance phase. In children, cardiovascular failure was as frequent as respiratory failure and occurred equally during induction, maintenance and recovery. Complications included airway management (*N* = 5), complications of intubation (*N* = 4), aspiration (*N* = 4), postoperative respiratory depression (*N* = 2), equipment failure (*N* = 1), halothane overdose (*N* = 2), anaphylactoid shock (*N* = 3), acute pulmonary edema (*N* = 2), severe arrhythmia (*N* = 2) and cardiac arrest with no obvious cause (*N* = 2). The incidence of complications increased with increasing American Society of Anesthesiologists (ASA) physical status, prior history of anesthesia, emergent procedures and preoperative fasting of less than eight hours [6].

### 2.2. General Pediatric Perioperative Adverse Events in the Modern Era

Several more recent studies analyzed the incidence and associations of pediatric perioperative adverse advents. Schleelein et al. at Children’s Hospital of Philadelphia analyzed non-cardiac cases for adverse events requiring a rapid response from April 2010 to September 2012. There were 213 events for a prevalence of 0.0046 (1:234) [7]. Respiratory events, primarily laryngospasm, were most common followed by cardiovascular events. Median age was lower in the event cohort than in controls (2.86 years vs. 6.20 years). Odds ratios for ASA physical status, multiple services involved in procedure, NORA vs. operating room location, and attending anesthesiologist experience were all significant. 

MacLennan and Smith analyzed critical events in the UK national reporting system from 2006 to 2008. Out of 606 incidents, six deaths were reported and 48 events resulted in severe harm [8]. The most common issues were related to medication errors (35.6%) with duplicate dosing in the OR and ward being the most notable. Airway and ventilation issues made up 18.8% of events, cardiovascular incidents 5.9%, and equipment issues (failure or unavailability) were involved in 15.7% of events. Communication and organizational issues were cited in 8.6% of reports.

The Anaesthesia Practice In Children Observational Trial (APRICOT) study was a prospective observational multicenter cohort study of severe critical events in pediatric anesthesia from 261 hospitals in Europe. From April 2014 to January 2015, 31,127 anesthetic procedures in 30,874 children were analyzed. The incidence of severe perioperative critical events was 5.2%. The incidence of respiratory events was 3.1% and cardiovascular events was 1.9%. The thirty-day in-hospital mortality rate was 10:10,000 and was independent of anesthesia type. Major risk factors included age under one year, complex medical history, and physical condition. A beneficial effect was noted with years of experience of the most senior anesthesia team member for respiratory and cardiovascular events rather than the type of institution or providers [9].

Boston Children’s Hospital analyzed 193 emergent (STAT) calls for help by the perioperative team between August 2011 and September 2015. They found that higher ASA status, history of respiratory illness, cardiac inciting event, occurrence during induction phase, post anesthesia care unit location, and calls initiated by an attending physician or fellow were more likely to be associated with a complicated STAT call. A subset of 108 calls indicated that age <1 year old and a history of prematurity were independent predictors of higher incidence of STAT calls [10].

## 3. Pediatric Anesthesia Safety Topics

### 3.1. Airway Events

Perioperative respiratory adverse events are a cause of major morbidity and mortality, contributing to 30% of perioperative cardiac arrests, and can lead to increased length of stay, worsened outcomes, and higher hospital costs [11,12]. Risk factors include age less than 6 years, a recent (<4 weeks) or currently active upper respiratory tract infection (URI), pulmonary comorbidity (asthma, prematurity, cystic fibrosis, etc.), current significant infectious disease, snoring, type of airway device used intraoperatively, experience of the anesthesiologist, and type of surgery [13]. 

A systematic review by Porter et al. of respiratory and hemodynamic perioperative adverse events in intravenous (IV) vs. inhalational induction in pediatric patients resulted in two studies that found no difference and two studies that found a higher risk of respiratory events with inhalation induction [14]. Hasani et al. showed no difference in coughing or laryngospasm with induction type [15]. Guard et al. showed no difference in coughing with sevoflurane vs. propofol, and neither group had bronchospasm or hypoxia. Laryngospasm did occur twice in the sevoflurane group and not at all in the propofol group, so a relative risk could not be calculated [16]. Chen et al. found a lower risk of coughing during emergence in the propofol group [17]. Ramgolam et al. found a higher risk of respiratory events in the inhalation group during induction and higher risk of perioperative respiratory adverse events as compared to the IV induction group [18]. More events occurred in children with multiple risk factors for respiratory adverse events (reactive airway disease or passive smoke exposure); however, this did not reach statistical significance [14]. Reasons for decreased respiratory events with an IV induction may be that muscle relaxation is given sooner, propofol may influence position of vocal cords (abduction), or propofol may suppress laryngeal reflex response. By comparison, sevoflurane maintains the airway in an excitement stage longer, potentially predisposing to laryngospasm.

WUS is a patient safety organization that collects data on perioperative anesthesia adverse events from multiple member institutions. Pfaff et al. analyzed WUS data on perioperative aspiration events in children from 29 institutions from 2010–2017. Out of 2,440,810 anesthetics, there were 135 pulmonary aspiration events (0.006%) with 110 cases (82%) resulting in escalation of care, 51 (38%) resulting in harm, and 2 (1.5%) resulting in death. Emergency surgery (14 of the cases) and higher ASA (≥3) status were more likely to experience aspiration. Reported causes of aspiration included gastrointestinal comorbid conditions (19%), post coughing event or laryngospasm (14%), nil per os (NPO) violation (11%), blood or secretions in the airway during or following the procedure (6%), and oral premedication reaction (3%) [19].

URIs are the most common perioperative comorbidity in children, and approximately 30% of children presenting for surgery have a URI [20,21]. Despite increased risk of respiratory events, most are without long-term sequelae, but there have been at least two peri-operative deaths in patients with URI [22,23]. The decision on whether to proceed with surgery in a child with an active URI is a long-standing debate. Subramanyam developed and validated a risk prediction tool in pediatric ambulatory anesthesia [24]. Lee et al. reported on perioperative respiratory adverse event risk in children with URIs and validated the current signs and symptoms, onset of symptoms, lung disease, device to be used for airway management and surgery type (COLDS) score as a potential algorithm to decide on whether to proceed or delay elective surgery. In the COLDS acronym, C stands for current signs and symptoms; O stands for onset of symptoms; L stands for lung disease; D stands for the device to be used for airway management; and S stands for surgery type (airway vs. non-airway) [13,25]. Another group performed a systematic review and meta-analysis on laryngeal mask airways (LMA) vs. other airway devices used in children with a URI. Five randomized clinical trials (RCTs) were included and found no statistical difference between LMA and endotracheal tube (ETT) regarding breath holding or apnea, laryngospasm, and arterial desaturation; however, the LMA did reduce cough compared with an ETT [26].

### 3.2. Cardiovascular Events

Another analysis of WUS data found 531 perioperative cardiac arrests out of 1,006,685 anesthetics from January 2010 to December 2015. Cardiac arrest was associated with age <6 months, ASA status III-IV, and emergency status. Increased mortality after cardiac arrest was noted with emergency status and off-hours (nights and weekends) procedures [27]. The APRICOT study reported 10 episodes of cardiac arrest in nine out of 30,874 patients (0.03%). Hypoxemia was cited as the cause in four cases, low cardiac output in four cases and hypotension in two cases [9]. 

An additional area of interest in pediatric anesthesia is the risk associated with children with congenital heart disease (CHD) who present for non-cardiac procedures. Taylor and Habre looked at National Surgical Quality Improvement Program (NSQIP) data and found that patients with single ventricle physiology, severe/supra-systemic pulmonary hypertension, complex lesions, and cardiomyopathy with reduced ventricular function were at increased risk of adverse events [28]. Lee et al. analyzed 3010 patients with CHD who presented for non-cardiac procedures over a five-year period. They found an 11.5% incidence of cardiovascular events and 4.7% incidence of respiratory events. Perioperative cardiovascular events were associated with an ASA status of 3 or higher, emergency cases, major and severe CHD, single ventricle physiology, and ventricular dysfunction. Orthopedic surgery, general surgery, neurosurgery, and pulmonary procedures were the most commonly associated with cardiac events. Respiratory events were associated with an ASA status of 4 or higher, and otolaryngology, gastrointestinal, general surgery, and maxillofacial procedures. They found that the incidence of cardiac events was higher than respiratory events, and though cardiovascular events were associated with cardiovascular status, respiratory events were not [29]. Institutions should consider the need for and availability of specialty trained pediatric cardiac anesthesiologists for complex congenital heart disease patients.

### 3.3. Medication Errors 

WUS data from 2010 to September 2016 from 32 institutions was analyzed for medication related events. The query included 2,316,635 anesthetics with 2087 adverse events reported. There were 276 medication errors reported, and this was the third leading cause of adverse events following respiratory and cardiovascular events. The medication errors most commonly involved opioids and sedatives/hypnotics. Thirty errors occurred during preparation, 67 during prescribing and 179 during administration. The most common error was administration of the wrong dose (*N* = 84), followed by administration of the wrong syringe (*N* = 49). Twenty-one percent (*N* = 57) of medication errors involved infusions as opposed to one-time bolus administrations. Errors were committed by all types of anesthesia providers, but most commonly by attendings. Over 80% of reported errors impacted the patient and more than half of those caused harm. Five percent (*N* = 15) of events required life-sustaining intervention. Ninety-seven percent of the errors were deemed likely or certainly preventable [30].

### 3.4. Handovers/Communication Errors

An additional analysis of WUS data reviewed complications associated with the anesthesia transport of pediatric patients. Of 2971 events, 148 events (5%) were related to transport and were primarily respiratory in nature. Almost 40% of events occurred in infants six months of age or younger. Almost 60% of the events were deemed at least somewhat preventable and over 36% were associated with harm. There were 86 reported cardiac arrests, 50 of which had respiratory causes, and 74% of those were related to anesthesia or perioperative team factors. Respiratory events occurred at all stages of care with 21.4% during preoperative transport and 75.5% postoperatively. Of unplanned extubations during transport, 93% occurred in patients six months old or younger. There were 10 medication events and two of those resulted in cardiac arrest. Root causes were attributed to provider and patient factors and, occasionally, verbal miscommunication was referenced [31].

### 3.5. Non-Operating Room Anesthesia (NORA)/Sedation

Uffman et al. reviewed WUS data from 2010 to 2015 to analyze severe outcomes of pediatric perioperative events occurring in operating rooms compared to off-site anesthetizing locations. There were 1594 events, of which 362 were associated with NORA locations. Adverse events in cardiac catheterization suites had higher odds of severe outcome compared to the operating room, but this difference was not found in other off-site locations [32]. However, other groups have reported a lower incidence of critical events at off-site locations compared to the operating room [9,10].

Lee et al. reviewed media reports of deaths associated with pediatric dental sedation and general anesthesia. Most deaths occurred among two- to five-year-olds (21/44) in an office setting (21/44) with a general/pediatric dentist (25/44) as the sole anesthesia provider. In the 25 deaths associated with a dentist providing the anesthetic, 17 were linked to a sedation anesthetic. Eleven cases were reviewed by an external body, and deficient practice patterns from these reports fell into the following categories: medication/dosing error (inappropriate doses of a sedative, a local anesthetic or a paralytic agent contributing to or resulting in cardiac arrest), inadequate monitoring (untrained staff who did not recognize respiratory failure in the post anesthesia care unit (PACU), and absence of vital sign monitoring, equipment or documentation), inadequate resuscitation (failure to recognize cardiac arrest, inadequate or no resuscitation efforts, limited resuscitation efforts due to untrained staff or inadequate equipment), and inadequate preoperative preparation (inadequate discussion of anesthetic risks and lack of medical evaluation). Eight adverse judgments concerned care in an office, while only one adverse judgment was associated with hospital/surgery center care [33].

Other pediatric subspecialists provide procedural sedation outside of the operating room. Kamat et al. prospectively collected data from 2007 to 2018 to analyze patient characteristics, medications, types of providers, adverse events, and interventions. A total of 432,842 encounters were included and divided into three 4-year epochs. Their findings included a decreasing trend in infants less than three months of age receiving sedation, a large increase in pediatric hospitalists providing procedural sedation, and a decrease in sedation provided by providers not trained in emergency medicine, critical care, or anesthesiology. In regard to medications used, they noted an increasing trend in the use of dexmedetomidine and a decreasing trend in the use of chloral hydrate and pentobarbital. Serious adverse events showed a non-significant increase overall [34].

### 3.6. Obesity

The global prevalence of childhood overweight and obesity increased from 4.2% in 1990 to 6.7% in 2010 and is expected to reach 9.1% or approximately 60 million children in 2020. In North America, 30% of school-age children are overweight or obese and 15% are obese [35]. Perioperative respiratory adverse events occur more commonly in obese children with significant associations between obesity and hypoxemia, upper airway obstruction, and difficult bag–mask ventilation [36]. Train et al. found that anesthesia and operative times were significantly longer for obese patients undergoing most types of surgical procedures [37]. The prevalence of obstructive sleep apnea (OSA) has increased with the increasing prevalence of obesity. Kako et al. reported increased PACU length of stay in 30% of cases and supplemental oxygen use was higher in patients with documented or likely OSA [38].

There are several preoperative considerations that must be assessed. The ASA practice guidelines recommend that questions regarding OSA (snoring, apnea, frequent arousals, morning headaches, and daytime sleepiness) be included in the preoperative interview for all obese patients over the age of one year. Obese patients with significant episodes of desaturation (<70% saturation) are at risk of ventricular dysfunction and are predictive of pulmonary hypertension in patients with OSA. Pre-operative echocardiography and electrocardiogram may be indicated. Special attention must be paid during the airway evaluation due to possible difficult mask ventilation and intubation [39].

Drug dosing in obese pediatric patients has several considerations. The volume of distribution, which determines loading dose and onset of action, may be altered in severely obese children. Drug clearance, which determines a drug’s maintenance dose, may be altered in obese patients, but this is not conclusive. Considerations for specific anesthetics in obese pediatric patients can be seen in Table 1.

In addition, several postoperative considerations must be recognized. Unplanned hospital admissions and length of stay in the PACU are higher in obese children. Although there are no specific guidelines on postoperative monitoring in obese patients, the ASA Task Force on Perioperative Management of Patients with OSA has recommended that patients should establish that they are able to maintain their baseline oxygen saturation during an unstimulated environment (preferably while asleep) [39]. Close monitoring is recommended with opioid use postoperatively and use of non-opioid analgesics, pain adjuncts, and regional techniques should be strongly considered.

### 3.7. Neonatal Intensive Care Unit Patients

Neonatal patients are at particular risk in the perioperative period given their size, fragility and physiology yet are vastly understudied in the perioperative arena. Rates of adverse events may be up to eight times higher for NICU patients compared to hospitalized adults [44]. According to 2012 National Surgical Quality Improvement Program Pediatric (NSQIP-P) data, neonates represent only 6% of all patients, yet account for 60% of 30-day post-operative morbidity and 16% of 30-day post-operative mortality [45]. Long et al. characterized adverse events in neonatal intensive care unit patients who recovered in the PACU from June 2014 to May 2018. Of 707 operative cases, 81 events were recorded and 64 of those were considered major events, all of which were respiratory in nature. The risk of any postoperative event was 11.5%, major respiratory event requiring intervention was 9.1%, and reintubation was 0.8%. Birth weight <1.58 kg and post gestational age at surgery <41 weeks were strongly associated with increased risk of major postoperative respiratory event. A patient with both those features had a sevenfold increase in the odds of a major respiratory event in the PACU [46].

France et al. conducted a prospective observational study of neonatal surgical patients to compare the incidence, severity, preventability and contributing factors of non-routine events (NREs) associated with perioperative handovers. An NRE is described as any deviation from optimal care based on the clinical situation. They found that 101 out of 130 cases (78%) had a clinician-reported NRE. They also evaluated direct handovers (handovers between the OR and NICU teams when the patient was retrieved from and/or returned directly to the NICU) and indirect handovers (between NICU nurse and pre-operative or PACU nurse). They found no difference in NRE rate between direct and indirect handovers. NRE severity and incidence of major morbidity as defined by NSQIP-P data were both significantly higher in the direct handover group, but is likely due to direct handovers involving more critically ill patients. They determined that nearly half of NREs resulted from preventable factors. Additional work continues in this area [47].

### 3.8. Neurotoxicity

Potential anesthetic neurotoxicity has been a topic of discussion in the realm of pediatric anesthesia for multiple years. The U.S. Food and Drug Administration (FDA) issued a safety announcement in 2016 stating that children under the age of three who undergo anesthesia for more than three hours or are exposed to repeated anesthetics could be at increased risk of learning, memory, or behavioral problems in the future. Because of this, Strategies for Mitigating Anesthesia-Related Neuro-Toxicity in Tots (SmartTots), a public–private partnership formed to identify, support, and fund pediatric anesthesia research focused on potential neurotoxicity. Through this partnership, studies such as the General Anesthesia Spinal (GAS) and the Pediatric Anesthesia Neurodevelopmental Assessment (PANDA) were undertaken. The GAS study compared general and spinal anesthesia in infants for urologic surgery and found that children under age two who were exposed to less than one hour of general anesthesia had no increased risk of adverse neurodevelopmental outcomes compared to infants who received a spinal anesthetic [48]. The PANDA study found that a single anesthetic exposure before age three did not increase the risk of neurocognitive dysfunction [49]. The Mayo Anesthesia Safety in Kids (MASK) Study did not find associations between anesthesia exposure before age three and general intelligence. Secondary outcomes indicated that multiple exposures may change some neuropsychological domains associated with learning and behavioral difficulties [50]. Based on the findings and the FDA’s warning, current recommendations are to not delay surgery and anesthesia for life-threatening conditions including but not limited to congenital heart defects, esophageal atresia, bowel obstruction, gastroschisis, omphalocele, diaphragmatic hernia, congenital lung lesions, pyloric stenosis, craniofacial reconstruction, and complex urological reconstruction. For elective procedures, parents should discuss the timing of surgeries that could be delayed without jeopardizing their child’s health. Since no anesthetic or sedative medication has been shown to be safer than any other, no specific alteration of anesthesia practice has been recommended [51].

### 3.9. Pediatric Anesthesia Patient Safety Resources

The SPA “advances the safety and quality of anesthesia care, perioperative care, and pain management in children by educating clinicians, supporting research, and fostering collaboration among clinicians, patient families, and professional organizations worldwide”. SPA also supports several committees including the Quality and Safety Committee whose goals include promoting an environment of process improvement, identifying key quality issues in pediatric anesthesia, and planning and implementing changes and projects. Further description is provided in the parallel manuscript to this, also by our group: “Pediatric Anesthesia Specialty Societies and Multi-Institutional Collaborations” [52]. One such project was the development of the Pedi Crisis checklist and mobile app [53]. The checklist was designed to support clinicians during pediatric perioperative life-threatening events. They have the latest evidence-based and expert opinion-based information in a just-in-time format and have been translated into multiple languages. The checklist has been used for both provider learning and just-in-time patient care, particularly among rural practitioners [54]. A COVID Pediatric Airway Checklist has also been developed.

SPA also sponsors WUS, a multi-institutional pediatric anesthesia quality improvement initiative and a designated Patient Safety Organization. Through its 37 participating institutions, data on thousands of pediatric anesthetics is collected each year and members have used this registry of serious adverse events to publish numerous papers and disseminate best practices. WUS also provides education in education science, holds twice-yearly meetings and monthly conference calls for members. One focus of WUS is quality improvement (QI). In fact, QI was used to design and implement WUS in 2008 [55]. Tjia et al. further describe the background and development of WUS [56].

## 4. Conclusions

Pediatric anesthesiology has made great strides in patient perioperative safety over the past several decades. With the recognition and development of subspecialty organizations such as SPA and its associated committees, associations, and special interest groups such as the SPA Quality and Safety Committee and WUS, the field of pediatric anesthesiology has become more focused on patient safety research to improve anesthetic care for pediatric patients by investigating adverse events. This includes investigating airway/respiratory events, cardiovascular events, medication errors, handover communications, NORA anesthesia and sedation, obesity, NICU patients, and potential neurotoxicity risk. This has encouraged improved perioperative adverse event reporting and analysis as well as an increase in QI initiatives. One recent QI initiative aims to decrease distractions on anesthesia providers during critical periods of anesthesia, and saw a 45% decrease with implementation of QI methods [57]. With increased event reporting and analysis, we can better investigate incidences, causes, and risks of complications and perioperative adverse events. Other areas for future research include resuscitation protocols, peri-operative nursing training, use of standardized protocols, enhanced recovery after surgery (ERAS), use of electronic medical record alerts, and many others. Continued and improved event reporting will continue to move pediatric anesthesiology forward in improving the safety of care for pediatric patients.

## Figures and Tables

**Table 1:** Considerations for anesthetic agents in pediatric patients with obesity.

Agent	Implication
Propofol	Obese children require lower weight-based dose of propofol for induction of anesthesia compared to normal-weight children (2 mg/kg vs. 3.2 mg/kg) [40].
Inhalational agents	Clinically insignificant difference with Isoflurane, Sevoflurane shows a faster recovery [41].
Succinylcholine	TBW (total body weight) should be used for dosing [41].
Non-depolarizing muscle relaxants	Vecuronium shows prolonged effect, may be due to TBW dosing. Rocuronium shows slight prolongation [41]. Ideal body weight (IBW) should be considered for dosing [39].
Opioids	Clearance of fentanyl is significantly increased, lower plasma concentration during early phase. Sufentanil clearance is similar [41]. Morphine, in contrast, is hydrophilic and should be dosed by TBW [39].
Benzodiazepines	Midazolam shows lower concentrations with obese patients and increased duration of action [42].
Dexmedetomidine	May decrease opioid requirements. No studies of dexmedetomidine pharmacology in pediatric obesity [41].
Ketamine	May decrease opioid requirements. Lipophilic but limited pharmacokinetic studies in obesity [39].
Acetaminophen	IBW should be used for dosing [43].
